# Kuru: A Journey Back in Time from Papua New Guinea to the Neanderthals’ Extinction

**DOI:** 10.3390/pathogens2030472

**Published:** 2013-07-18

**Authors:** Pawel P. Liberski

**Affiliations:** Department of Molecular Pathology and Neuropathology, Medical University of Lodz, Kosciuszki st. 4, Lodz 90-419, Poland; E-Mail: ppliber@csk.umed.lodz.pl; Tel./Fax: +48-42-679-14-77

**Keywords:** kuru, prion diseases, neuropathology, D. Carleton Gajdusek

## Abstract

Kuru, the first human transmissible spongiform encephalopathy was transmitted to chimpanzees by D. Carleton Gajdusek (1923–2008). In this review, I briefly summarize the history of this seminal discovery along its epidemiology, clinical picture, neuropathology and molecular genetics. The discovery of kuru opened new windows into the realms of human medicine and was instrumental in the later transmission of Creutzfeldt-Jakob disease and Gerstmann-Sträussler-Scheinker disease as well as the relevance that bovine spongiform encephalopathy had for transmission to humans. The transmission of kuru was one of the greatest contributions to biomedical sciences of the 20th century.

## 1. Introduction

**Figure 1:**
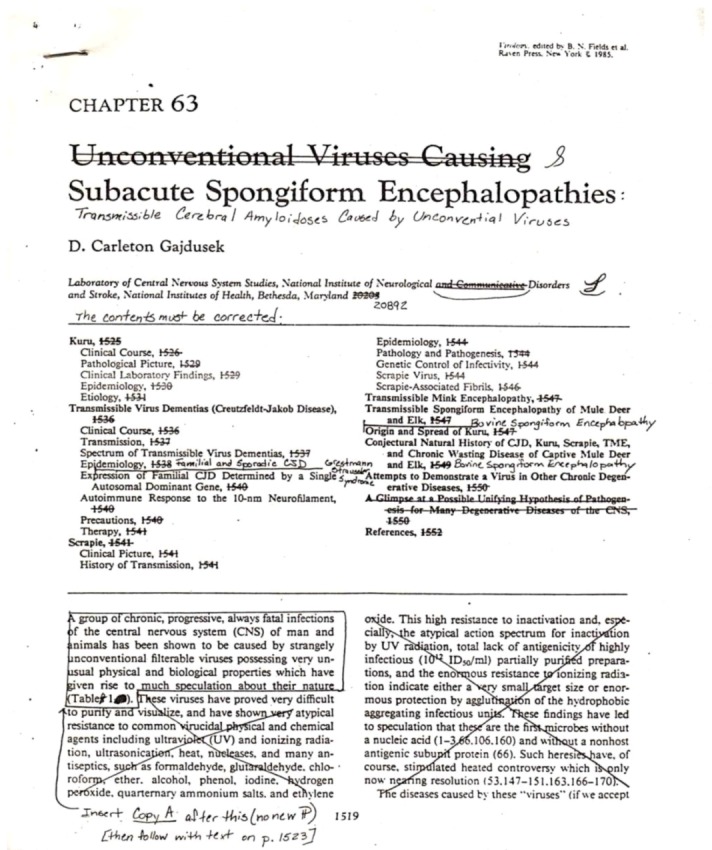
A copy of a galley D. Carleton Gajdusek performed for his last chapter in the Fields Virology [23].

Kuru is a disease that will forever be linked with the name of D. Carleton Gajdusek [1,2,3,4,5,6,7,8,9,10,11]. It was the first human prion disease transmitted to chimpanzees and subsequently classified as a transmissible spongiform encephalopathy (TSE), or slow unconventional virus disease. It was first reported in Western medicine in 1957 by Gajdusek and Vincent Zigas (Figure 1) [12,13]. 

The recognition of kuru as a neurodegenerative disease that is transmissible (*i.e.*, infectious is a microbiological term) [14,15,16,17,18] and subsequent transmission of Creutzfeldt-Jakob disease (CJD) [19] not only proved that kuru is not merely an exotic disease caused by cannibalism on a remote island, but it represents a novel class of diseases caused by a novel class of pathogens. Kuru won D. Carleton Gajdusek a Nobel Prize in 1976 and subsequently contributed to another Nobel Prize awarded to Stanley B. Prusiner in 1997. Indirectly, kuru was linked to a third Nobel Prize for Kurt Wüthrich, who determined the structure of the prion protein [20]. As Gajdusek stressed for the last time in his life at the Royal Society meeting on kuru [21], the solving of the kuru riddle contributed to developing ideas of molecular casting and to further understanding such diverse areas as dermatoglyphes and osmium shadowing in electron microscopy amyloid-enhancing factors. Recently, kuru research has impacted the concepts of nucleation-polymerization and led to a concept of “conformational disorders” [22,23,24] or prionoids [25]. 

## 2. Background and Ethnographic Setting

**Figure 2:**
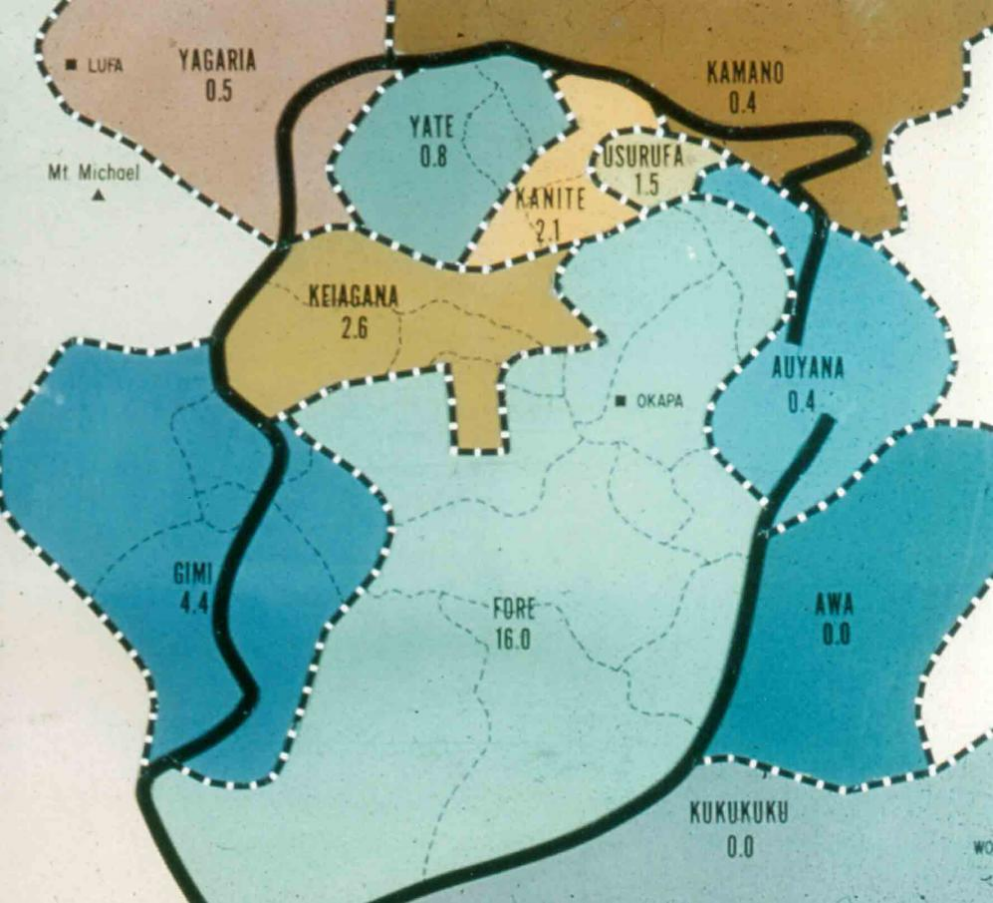
A map of the kuru region with prevalence of the disease in different linguistic groups. Courtesy of late D. Carleton Gajdusek.

**Figure 3:**
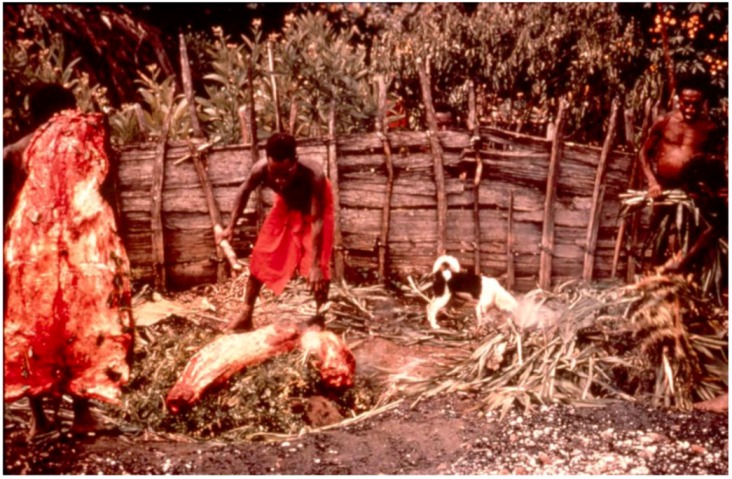
An example of Fore people. Courtesy of late D. Carleton Gajdusek.

“Kuru” in the Fore language of Papua New Guinea (Figure 2, Figure 3) means to tremble from fever or cold [13,26,27,28,29,30,31,32,33,34,35,36,37,38,39]: “The natives of almost all of the Fore hamlets have stated that it has been present for a “long time”; but they soon modify to mean that in recent years it has become an increasingly severe problem and that in the early youth of our oldest informants there was no kuru at all” [13]. 

Kuru was restricted to natives of the Foré linguistic group in Papua New Guinea’s Eastern Highlands and neighboring linguistic groups (Auiana, Awa, Usurufa, Kanite, Keiagana, Iate, Kamano, Gimi; Figure 2). Those groups into which kuru-affected peoples did not intermarry, such as the Anga (Kukukuku), separated from the Fore by Lamari River and the remote Iagaria, Kamano and Auiana people, were not affected. Zigas and Gajdusek [40,41] noticed that when Fore of Kasarai from the South Fore moved temporarily to live with the Yar people and settled there for about a decade, they still had kuru cases. It seems that kuru first appeared at or shortly after the turn of 20th century [42,43,44,45] in the Uwami village of Keiagana people and spread from there to the Awande in the North Fore where the Uwami had social contacts. Within 20 years it had spread further into the Kasokana (in 1922 according to Lindenbaum [44]) and Miarasa villages of North Fore, and a decade later had reached the South Fore at the Wanikanto and Kamira villages. The slow march of kuru was inconsistent with a contemporary genetic hypothesis of Bennett *et al*. [46,47] but it was consistent with a slow infectious disease. Kuru became endemic in all villages which it entered and became hyperendemic in the South Fore region. All native informants stressed the relatively recent origin of kuru. Interestingly enough, when kuru first appeared, it was considered poetically by Foré as similar to “the swaying of *casuarinas tree*” and kuru was labeled *cassowary disease* to stress the similarity between *cassowary* quills and “waving *casuarinas* fronds”. 

## 3. Cannibalism

Ritualistic endocannibalism (eating of relatives as part of a mourning ritual in contrast to eating enemies, *i.e.*, exocannibalism) was practiced not only in the kuru area but in many surrounding Eastern Highland groups in which kuru never developed [45,48,49,50,51,52,53]. “*When a body was considered for human consumption, none of it was discarded except the bitter gall bladder. In the deceased’s old sugarcane garden, maternal kin dismembered the corpse with a bamboo knife and stone axe. They first removed hands and feet, then cut open the arms and legs to strip the muscles. Opening the chest and belly, they avoided rupturing the gall bladder, whose bitter content would ruin the meat. After severing the head*, *they fractured the skull to remove the brain. Meat, viscera, and brain were all eaten. Marrow was sucked from cracked bones, and sometimes the pulverized bones themselves were cooked and eaten with green vegetables. In North Fore but not in the South, the corpse was buried for several days, then exhumed and eaten when the flesh had “ripened” and the maggots could be cooked as a separate delicacy”*[45].

The first European who witnessed kuru was Ted Ubank, a gold prospector, in 1936 [45]. In the late 1930s and 1940s, many gold miners, Protestant missionaries, and government officials became familiar with the presence of endocannibalism of Eastern Highland tribes. In early 1951 and 1953, kuru was observed by a pair of anthropologists Berndt and Berndt [45], and the first mention of kuru (*skin-guria* in Pidgin) was included in reports of patrol officers in 1953. Zigas was told about kuru in 1955 and he was joined by DC. Gajdusek two years later. I asked Gajdusek in the 2000s when the hypothesis of cannibalism as a vehicle to spread kuru was first envisaged. His response was that “*even completely drunk would come to the conclusion that a disease endemic among cannibals must be spread through eating corpses*”. Gajdusek said this some 50 years after the discovery of kuru. The first who formally published the hypothesis that kuru spreads through cannibalism was Lindenbaum and Glasse [48,53,54]. However, according to Gajdusek, the hypothesis of cannibalism was taken for granted but it is also true that in his Nobel Prize lecture he said that kuru spread by ‘*conjunctival, nasal and skin contamination with highly infectious brain tissue*”; thus, at that time Gajdusek was skeptical about cannibalism hypothesis which he regarded as exotic. In 1981 [29], Gajdusek wrote again “*It is useless to speculate about the origin of this idea; I know of few Europeans who did not arrive at such a conjecture. All the missionaries, traders, and government workers and their families in the Eastern Highlands knew that most of the indigenous people in that area had been cannibals […] eating close relatives in mourning rites,[…] At one rather casual stage of hypothesizing, we wondered whether cannibalism without infection might be involved by a mechanism of hypersensitivity. […] Robert Glasse has quoted this as the only mention of cannibalism he found in our publishing writing. That this should have been the case, however, indicates not the prior absence of idea, but rather its complete its complete obviousness.*” Some authors even denied the very existence of cannibalism [55] but the denial clearly belongs to another mythology. Probably the last episode of cannibalism took place in 1978 [44].

**Figure 4:**
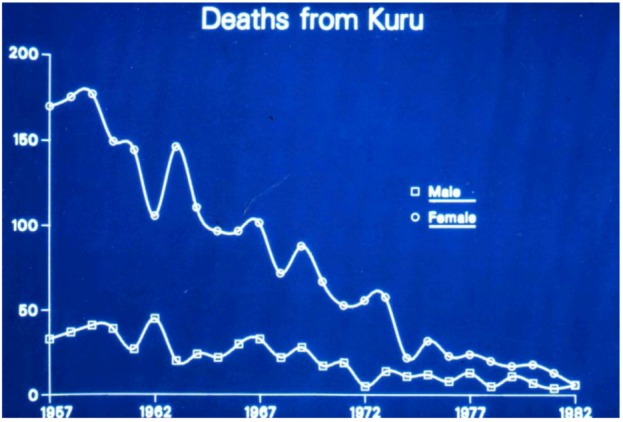
Deaths from kuru 1957–1982. Courtesy of late D. Carleton Gajdusek.

In subsequent years, the number of kuru cases has steadily declined (Figure 4), with the youngest patients becoming progressively older, and the disease is now extinct; however, we cannot be sure that, with an incubation period in excess of 50 years, a limited number of cases will not appear in the future.

Among the Foré, kuru was believed to result from sorcery [45]. The victim was chosen because of some real or imaginary faults [41,56]. To cause kuru, a would-be-sorcerer would need to obtain a part of the victim’s body (nail clippings, hair) or excreta, particularly feces- or urine-soaked vegetation, saliva, blood, or partially consumed food such as peelings from sweet potato eaten by the victim, or clothing (“maro”). These were packed with leaves and made into a “*kuru bundle*” and placed partially submerged in swampy land. Subsequently, the sorcerer shook the package daily until the tremor characteristic of kuru was induced in his victim. As a result, kinsmen of a kuru victim attempted to identify and subsequently kill (“tukabu”) a suspected sorcerer if they could not bribe or intimidate him to release a victim from the kuru spell.

Divination rituals helped to identify a sorcerer. One method was to collect water for the kuru victim from different sources; if one “induced” vomiting, it was considered to be near the sorcerer’s residence. Another method was to place hair clippings from a kuru victim into a bamboo cylinder, and a freshly killed possum in another cylinder. Calling the name of a suspected sorcerer while shaking the cylinders, a member of the victim’s family placed the possum-containing cylinder into a fire. The sorcerer was identified if the liver of the possum, believed to be the residence of his soul, remained uncooked. Still another rite was the roasting of small rats, each in a separate bamboo cylinder, each one having been given the name of a hamlet or village in which the suspected sorcerer lived. Careful inspection of the rat’s viscera helped to identify the sorcerer.

Killing of a sorcerer – *tukabu* – was a ritualistic form of vendetta; it included crushing with stones the bones of the neck, arm, and thigh, as well as the loins, biting the trachea, and grinding the genitalia with stones and clubs. As sorcerers were mostly adult men, whereas kuru victims were mostly women and children of both sexes, killing of male sorcerers contributed somewhat to maintaining the sex ratio in a population devastated by the kuru deaths of their women. Also, because kuru victims were mostly women, frequently nursing children, those children often died of malnutrition as Fore did not accept transferring the orphaned child to another woman.

## 4. Kuru Etiology – the Insight into A Novel Class of Pathogens

Although on epidemiological grounds the etiology of kuru was thought to be infectious, patients had no meningoencephalitic signs or symptoms (fever, convulsions, or coma), no cerebrospinal fluid pleocytosis or elevated protein level and, on autopsy, no perivascular cuffings or other signs of inflammatory brain pathology. Neither the environmental [57,58,59] nor then available genetic studies [27,60,61,62,63,64,65,66,67,68] provided any clues. Moreover, all attempts to transmit kuru to small laboratory animals or to isolate any microorganism including a virus, using tissue cultures or embryonated hen’s eggs were unsuccessful. In other wide ranging investigations, neither exhaustive genetic analyses nor the search for nutritional deficiencies or environmental toxins resulted in a tenable hypothesis [58,59]. 

On July 21, 1959, while in New Guinea, Gajdusek received a letter from the American veterinarian, William Hadlow, at the Rocky Mountain Laboratory in Hamilton Montana [35,69,70,71,72,73], which pointed out the analogies between kuru and scrapie, a slow neurodegenerative disease of sheep and goats known to be endemic in the United Kingdom since the XVIIIth century [74] and experimentally transmitted in 1936 [75,76]. Having seen kuru plaques at a Wellcome Medical Museum exhibition in London, he enclosed a copy of a letter pointing out this similarity to the editor of Lancet [69,72]. Hadlow based his observations not merely on the presence of amyloid plaques but mainly because of the presence of vacuolated neurons:

“*I’ve been impressed with the overall resemblance of kuru, and an obscure degenerative disorder of sheep called scrapie […] The lesions in the goat seem to be remarkably like those described for kuru. […] All this suggests to me that an experimental approach similar to that adopted for scrapie might prove to be extremely fruitful in the case of kuru. […] because I’ve been greatly impressed by the intriguing implication, I’ve submitted a letter to The Lancet.”*

**Figure 5:**
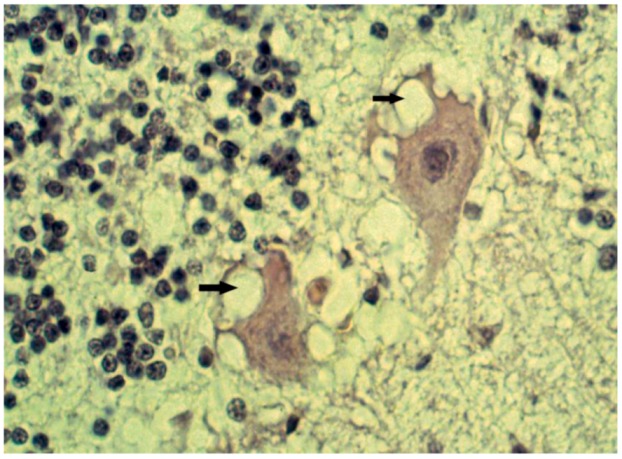
Intraneuronal vacuoles (arrows) in Purkinje cells. Courtesy of late D. Carleton Gajdusek.

A similar observation was made by a veterinary neuropathologist Innes [77] during his visit to the Gajdusek laboratory ([78]; Gajdusek – telephone conversation, 2008). Hadlow, in his recollection of that seminal observation pointed out intracellular vacuoles as those neuropathological changes that attracted his attention some forty years ago [79,80]. Those intracellular vacuoles in scrapie (Figure 5) were first described by Besnoit and his colleagues in 1898 [75]. Dr. Gajdusek replied that “*[As you may have been able to gather from our articles on kuru, we are pursuing the matter of possible infectious etiology extensively – I am, in fact, a virologist by training. However, we have thus far had poor luck with inoculation experiments and the possibility of doing more extensive inoculation works has, until now, been small. We are, however, proceeding accordingly at the present time and frozen and fresh material are being injected into a number of animal hosts during this year’s work on kuru. In your note to LANCET, which I am deeply grateful to you for bringing to my attention, I note that you have probably not seen our extensive pathological description of kuru which includes some features which were little stressed in the report you have quoted]*”, and took up Hadlow’s recommendation to hold small laboratory rodents and (especially) apes and monkeys for longer periods of observation than had been thus far been carried out. He also renewed attempts to obtain optimal tissue for inoculation from rapidly autopsied kuru patients (letter from DC. Gajdusek dated August 6th, 1959 [69]). 

**Figure 6:**
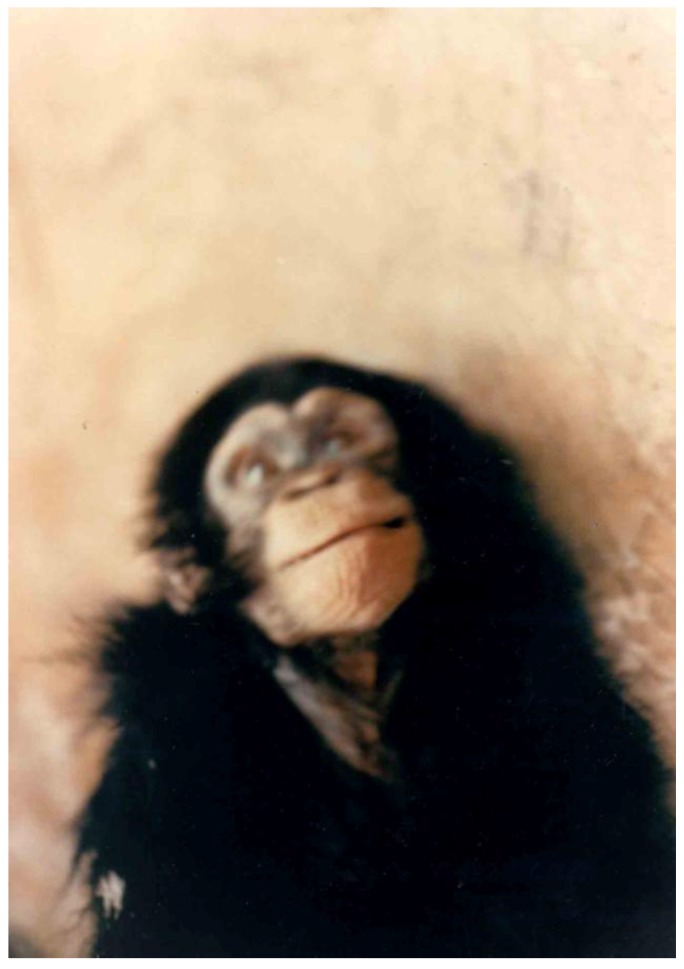
The first chimp affected with kuru. Courtesy of late C. J. Gibbs.

In 1961, Gajdusek presented a lecture at the Xth Pacific Science Congress in Honolulu entitled “Kuru: an appraisal of five years of investigation. With a discussion of the still undiscardable possibility of infectious agent” in which he said: “In spite of all the genetic evidence, both the pathological picture and the epidemiological peculiarities of the disease persistently suggest that some yet-overlooked, chronic, slowly progressive, microbial infection may be involved in kuru pathogenesis. Similar suspicion prevails in our current etiological thinking about a number of less exotic and less rare chronic, progressive degenerative diseases of the central nervous system in man. Thus, […], amyotrophic lateral sclerosis, Schilder disease,leukoencephalitis, Koshevnikoff’s epilepsy syndrome in the Soviet Union, the Jakob-Creutzfeldt syndromes, acute and chronic cerebellitis, and even many forms of Parkinsonism, especially the Parkinsonism dementia encountered among the Chamorro population in Guam, continue to suggest the possibility that in man there may be infections analogous to the slow infections of the nervous system of animals which were intensively studied by Bjorn Sigurdsson, the Icelandic investigator who formulated the concept of “slow virus infections” ”. This contention preceded the discovery of kuru transmissibility by more than 4 years [81]. Parenthetically, many of the diseases mentioned by Gajdusek are now grouped together under the umbrella of “protein conformational disorders” [22,24,82,83]. Finally, in 1965, in a monograph “Slow, Latent and Temperate Virus Infections” which resulted from a meeting convened in 1964, Gibbs and Gajdusek [84] wrote in an addendum “*although several of the inoculated primates died of acute infection during the period of observation, […] none has developed signs suggestive of chronic neurological disease until the recent onset in two chimpanzees (Figure 6). The first of these, inoculated 20 months previously with a suspension of frozen brain material from a kuru patient, has developed progressive incapacitating cerebellar signs with ataxia and tremor; the second, similarly inoculated with a suspension of brain material from another kuru patient, has developed, 21 months after inoculation, slight wasting lassitude, and some tremor which appeared to be progressive. Whether these syndromes are spontaneous or related to the inoculation remains to be determined*.”

## 5. Epidemiology of Kuru – A Strong Support of the Cannibalism Theory

Kuru incidence increased in the 1940 and 1950s [12,18,85,86,87,88] to approach the mortality rate in some villages 35/1000 among a population of some 12,000 Fore people [78,89]. This mortality rate distorted certain population parameters: in the South Fore, the female: male ratio was 1:1.67 in contrast to 1:1 ratio in unaffected Kamano people. This ratio increased to 1:2 to even 1:3 among South Fore. Gajdusek even calculated the women deficit in the population to be 1676 persons [36]. The almost total absence of kuru cases in South Fore among children born after 1954 and the rising of age of kuru cases year by year suggested that transmission of kuru to children stopped in the 1950s [90,91,92] when cannibalism ceased to be practiced among the Fore people. Also, brothers with kuru tend to die at the same age which suggested that they were infected at similar age but not at the similar time. The assumption that affected brothers were infected with kuru at the same age led to a calculation of minimal age of exposure for males to be in a range of 1 – 6 years with a mean incubation period of 3–6 years and the maximum incubation period of 10–14 years [42].

Alpers and Gajdusek wrote a year before the transmission of kuru was published [85] “*The still baffling, unresolved problem of the etiology of kuru in the New Guinea Highlands has caused as to wonder whether or not any or many of the unusual features of its epidemiological pattern and its clinical course may not be changing with time, or even altering drastically under the impact of extensive rapid cultural change, the result of ever increasing inroads of civilization upon the culture of the Fore people”.* This was indeed the case. The comparison of total number of deaths from kuru in the period 1961–1963 and 1957–1959 showed a 23% reduction, and among the children, 57% reduction and the kuru mortality rates dropped from 7.64 to 5.58 deaths per thousand. These alterations were not uniform, the North Fore reduction exceeded the South Fore reduction and it is worth recalling that the South Fore kuru deaths accounted for 60% of the total. This trend has tended to be observed until the disappearance of kuru epidemic [93].

The almost “formal” proof that kuru was indeed transmitted by cannibalism was provided by Klitzmann *et al*. [94] who studied clusters of kuru patients who participated in a limited number of kuru feasts in 1940s and 1950s. Three clusters were identified, one of which will be recalled here. Two brothers, Ob and Kasis from Awande village, North Fore developed signs and symptoms of kuru in 1975, 21 or 27 years after the latest or the earlier exposure, respectively. They participated in two feasts for kuru victims. In those feasts, the closes relatives were the major mourners actively participated in the consumption of the dead. 

Of interest, Klitzmann *et al*. [94] noticed that taking into consideration the fact that Fore women participated in numerous kuru feasts, it is strange that any of them survived into the 1970s. Modern molecular genetics explained this fact in terms of the codon 129 polymorphism of the *PRNP* gene. In the younger patients, homozygotes 129^Met Met^ predominate; the latter finding is reminiscent that of variant CJD (vCJD) [95] and suggests the increased susceptibility of 129^Met Met^ individuals, with a shorter incubation period than other *PRNP* codon 129 genotypes.

## 6. Transmission Experiments

**Figure 7:**
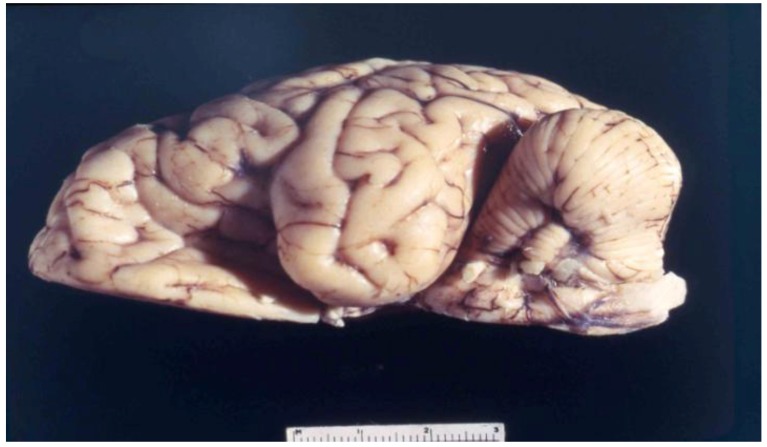
Macroscopic view of the chimp kuru; 67-10825-2, Courtesy of late D. Carleton Gajdusek.

The transmission of kuru to chimpanzee (Figure 6, Figure 7) won a Nobel Prize for Gajdusek in 1976 [14,16,37,38,81,96,97,98,99,100,101]. The list of non-human primates to which kuru was transmitted over the years is given in Table 1 and Table 2. They include Rhesus monkeys [102], marmosets [103], Gibbon and Sooty Mangabey monkeys [104]. The detailed description of experimental kuru in 41 chimpanzees was published in 1973 [2]. The incubation period varied from 11 to 39 months (the average, 23 months for the first passage; 12 months for the second passage; 13 months for the third passage and the same, for the fourth passage and the clinical course was divided into 3 stages:

1. Early stage (I)
  a) prodromal period characterized by earliest alterations in behavior; animals became inactive, sometimes “extremely dirty” and submissive. “*Vicious and aggressive animals became passive and withdrew from competition with their normal cagemates, allowing smaller chimpanzees to tease and take food from them”[..] periods of sullen apathy were often interrupted by outbursts of furious screaming”,*
  b) Period of minimal disabilities characterized by minor motor dysfunction; animals did not want to go outside cages, “to run or to climb”, they were slow and fell with forced movements; the movements were “*like [..]in slow-motion cinema”.*
2. Intermediate stage (II)
  The onset of this stage was characterized by difficulties exhibited when a chimp tried to rise from a supine position; gait became ataxic but animals still could sit. The gait of chimpanzees is *quadrupedal* – “knuckle walking” where animals placed hands on the ground not with palms but with knuckles and this pattern is preserved but the gait itself is grossly ataxic and dysmetric. Truncal titubation, so characteristic for human kuru, is present since stage II. Passive muscle tone is increased and flexion contractures may develop if an animal lives long enough. Severe coarse tremor is seen, choreiform movements are observed and the negligence develops. Difficulties in seeing, lateral nystagmus and intermittent left strabismus was seen. “Babinsky” sign was occasionally observed.
3. Late stage (III)
  Characterized by severe neurological deficits: they could not rise by themselves from a supine position, they could not sit but placed themselves in one position, and decubitus ulcers were common. They eat inedible objects. A severe startled response comprising flexion of all extremities accompanied by violent coarse trembling of all limbs was a characteristic finding.

Kuru neuropathology in chimpanzees was described by late Elisabeth Beck and Daniel [105,106,107,108,109,110,111]. The neuropathological picture was practically identical to that of natural kuru except for the absence of amyloid plaques. In the cerebral cortex, the spongiform change and intraneuronal vacuoles were the most prominent lesions, accompanied by a severe astrocytic gliosis. Binucleated neurons were prominent; the same type of neuronal lesions were also seen in the Spider monkey [112].

**Table 1:** Non-primate host range for kuru transmission; incubation period in months.

Species	Incubation period (months)
Goat (Capra hircus)	(104)+
Guinea pig (Cavia porcellus)	(27)
Opossum (Didelphis marsupialis)	(22+)
Domestic cat (Felis domesticus)	(59)
Gerbil (meriones unguiculatus)	(24)+
Hamster (Mesocricetus auratus)	(28)
Mous (Mus musculus)	22.5
Ferret (Mustela putorius)	18 – 70.5
Mink (Mustela vision)	45
Sheep (Ovis aries)	(63)+

Number in parenthesis – numer of months elapsed since the inoculation, during which the animals remained asymptomatic.

**Table 2:** A host range of the primates susceptible for kuru.

Species	Incubation period (months)
**Apes**	
Chimpanzee (Pan troglodytes)	10–82
Gibbon (Hylobates lar)	+ (10)
**New World Monkeys**	
Capuchin (Cebus albifrons)	10 – 92
Capuchin (Cebus paella)	11–71
Spider (Ateles geofffroyi)	10–85.5
Moramoset (Saguinus sp)	1176
Wolly (Lagothrix lagotricha)	33
**Old World Monkeys**	
African Green (Cercopithecus aethiops)	18
Baboon (Papio anubis)	(130)
Bonnet (Macaca radiate)	19–27
Bushbaby (Galago senegalensis)	(120)
Cynomolgus macaque (Macaca fascicularis)	16
Patas (Erythrocebus patas patas)	(136)
Pigtailed macaque (Macaca nemestrina)	70
Rgesus (Macaca mulatta)	15–102
Sooty mangabey (Cercocebus atys)	+(2)
Talapoin (Cecopithecus talapoin)	(1+)

## 7. Clinical Manifestations

“*I was still very young when I saw [kuru] and even after we treated it there was no help. Everyone was falling apart. [Kuru victims] were aware there was no cure and that they would die. It wasn’t just one person that this sickness came to – there were about three in a house line and then after they died there would be another three. It was…ongoing…there were many deaths. Once a [person]…was affected by kuru [their] family would think that the clan had poisoned [them] and they would start…shooting at each other and that made it worse. It was chaos ! (Taurubi)* .

[113]

**Figure 8:**
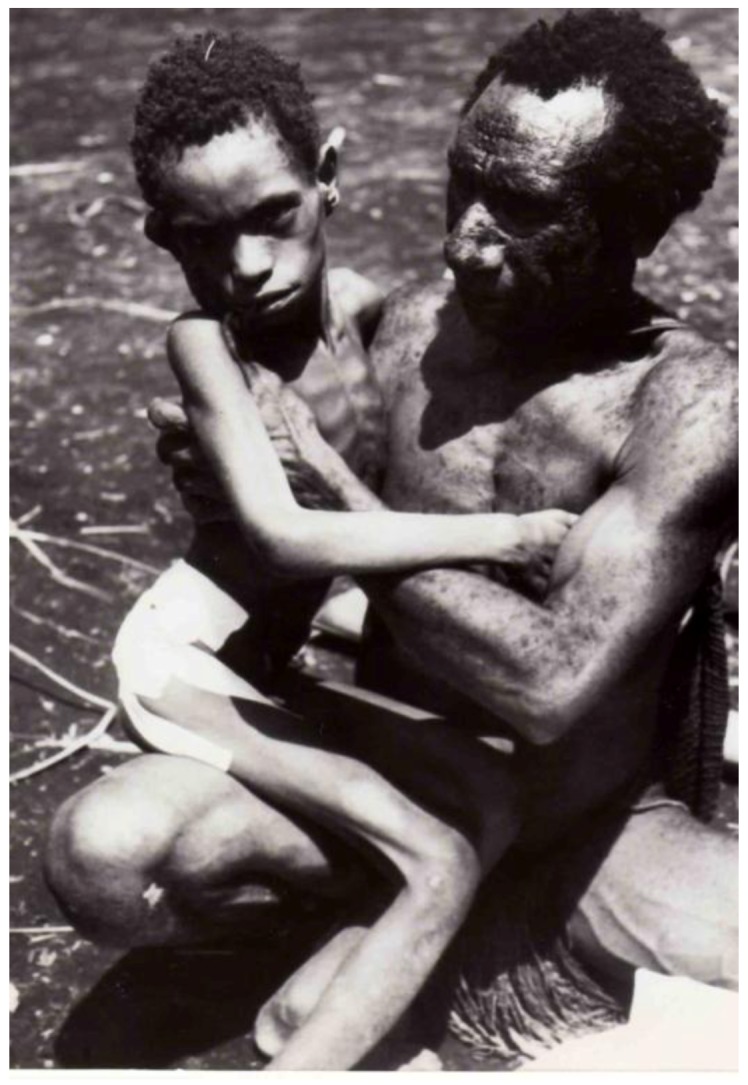
A young Fore girl in terminal stage of kuru, held by her father. Intravenous and tube feeding permitted her to live on to this advanced stage of disease at the Kuru Hospital, whereas in the village she would have been smothered by her relatives earlier, since her inability to swallow without aspiration of food or water would have caused her otherwise to starve and thirst to death. DCG-57-PNG-1136. Courtesy of late D. Carleton Gajdusek.

**Figure 9:**
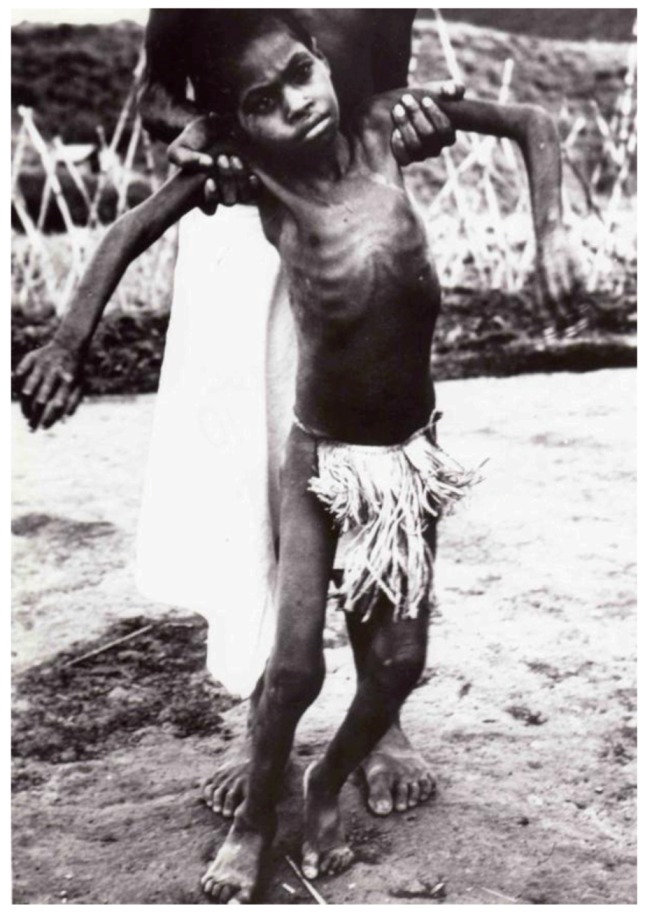
A child with advanced kuru who could neither stand nor sit without support. Courtesy of late D. Carleton Gajdusek.

**Figure 10:**
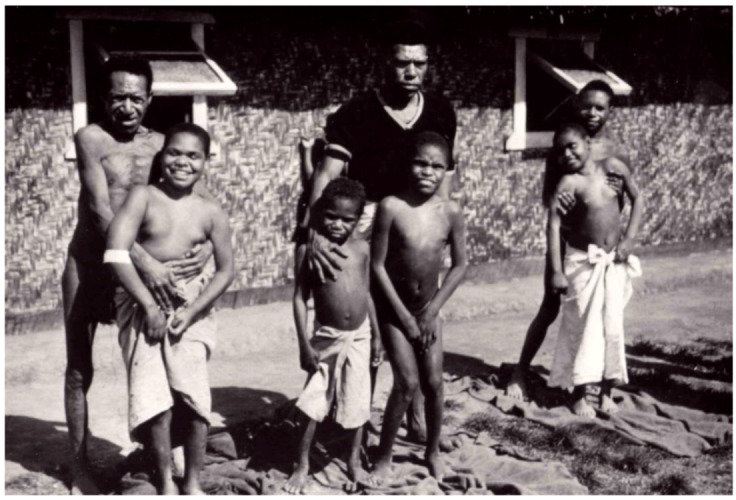
Three kuru patients at the kuru hospital in Okapa requiring support to stand erect. The Fore adolescent supporting the patient on the right, himself had incipient kuru and died within one year. The other two died within six months. Courtesy of late D. Carleton Gajdusek.

Kuru is an invariably fatal cerebellar ataxia accompanied by tremor, choreiform and athetoid movements (Figure 8, Figure 9, Figure 10) [18,28,32,35,74,113,114,115,116,117,118,119,120,121,122,123]. In contrast to the neuropathological picture, neurological signs and symptoms are highly uniform. Dementia typical for most subtypes of sporadic CJD, is barely noticeable, and if it is present, then only late during the evolution of the illness. In contrast, kuru patients often displayed emotional alterations, including inappropriate euphoria and compulsive laughter (the journalistic notorious “laughing death” or „laughing disease”), or apprehension and depression. Kuru is divided into three clinical stages: ambulant, sedentary and terminal (the Pidgin expressions, *wokabaut yet*; *i.e.*, is still walking”, *sindaun pinis*; *i.e.*, is able only to sit” and *slip pinis*; *i.e.*, is unable to sit up”) [114,124]. The duration of kuru, as measured from the onset of prodromal signs and symptoms until death was about 12 months (3 – 23 months) [114,124].

There is an ill defined prodromal period (*kuru laik i-kamp nau* –*i.e.*, “kuru is about to begin”) characterized by the presence of headache and limb pains, frequently in the joints; knees and ankles came first, followed by elbows and wrists; sometimes, interphalangeal joints were first affected; abdominal pains and loss of weight. This period lasted approximately a few months. Fever and other signs of infectious disease are never seen but the patient’s general feeling was reported as reminiscent of that accompanying acute respiratory infection. Some patients even said that they expected a cough to come and when it did not, they started to fear incoming kuru. 

The prodromal period is followed by the “ambulant stage”, the end of which is defined when the patient is unable to walk without a stick. The patients were psychologically supported by a community; one of the very important sign of this is to search for a sorcerer who, as already mentioned, they believed caused kuru. This period is characterized by the onset of subtle signs of gait unsteadiness that are usually only self-diagnosed, but which over a period of a month or so progress to severe astasia and ataxia. Incoordination of the muscles in trunk and lower limbs followed. As patients were well aware that kuru heralded death in about a year, they became withdrawn and quiet. A fine ‘shivering’ tremor, starting in the trunk, amplified by cold and associated with a “goose flesh”, is often followed by titubation and other types of abnormal movements. Attempts to maintain balance result in clawing of the toes and curling of the feet. Plantar reflex is always flexor while clonus, in particular ankle clonus but also patellar clonus, are hallmarks of the clinical picture, however, clonus may be only present for a limited period of time. The ankle clonus was in most cases the most enhanced, but patellar clonus and clonus of fingers and toes were also readily elicited. 

In the early stages ataxia could be demonstrated only when the patient stood on one leg; the Romberg sign was almost always negative (2 of 34 kuru cases in Alpers’ series [124]) but with the progression of disease, ataxia became marked and the Romberg sign became positive; indeed, the patient cannot stand with his feet close together. Ataxia in the upper limbs followed that in the lower limbs; dysmteria was usually the first sign of the upper limb ataxia. Intention tremor was found in 19 of 34 cases in Alpers series in the first stage of kuru but was constantly present in the second stage. Dystarthria appeared early. Resting tremor is a cardinal sign of kuru. According to Alpers “*[it is] difficult to describe and analyze. It appears to include the following components: a shivering component, an ataxic component, and, in the latter stages and certain cases only, the extrapyramidal component. A fine shivering-like tremor may be present from the onset of disease […] it is potentiated by cold and thus may not be found in the heat of the day; a sudden drop in temperature not sufficient to make others shiver will induce it in kuru patients. As ataxia increases a more obvious ataxic component is added and the shaking movements become wilder and more grotesque”.* The major component in kuru resting tremor, is the ataxic one: it is enhanced by the muscular activity and when the patient became motionless, it subsides; “*It often seemed to be triggered by minor movement, an adjustment of posture, stretching out the arm in greeting, or even a sudden turning of the eyes”.* Patients learn how to control tremor. A child trembling violently, described by Zigas and Gajdusek [40,41] found that he may almost completely abolish the tremor when curled into flexed, fetal position in his mother’s lap.

A horizontal convergent strabismus is a typical sign, especially in younger patients; nystagmus was common but the papillary responses were preserved. Facial hemispasm and supranuclear facial palsies of different kinds were also common. In one case, a transitory facial paralysis of upper motor neuron type was detected.

The second „sedentary” stage begins when the patient is unable to walk without constant support and ends when he or she is unable to sit without it. “*The gait was, by definition non-existent. However, if a patient was ‘walked’ between two assistants a caricature of walking was produced, with marked truncal instability, weakness at hips and knees and heavy leaning on one or other assistant for support; but steps could be taken, and were characterized by jerky flinging, at times decomposed movements, which led to a high-steppage, stamping gait’.* Postural instability, severe ataxia, tremor and dysarthria progress endlessly through this stage. Deep reflexes may be increased but the plantar reflex is still flexor. “Jerky ocular movements: were characteristic. Opsoclonus, a rapid horizontal ocular agitation was also occasionally noticed. Zigas and Gajdusek [40,41] reported a peculiar, jerking, clonic movements of the eyelids and eyebrows in patients confined to the dark indoors of huts and then transported outdoors to the light. Two cases of 34 showed signs of dystonia.

In the third stage, the patient is bedridden and incontinent, with dysphasia and primitive reflexes, and eventually succumbs in a state of advanced starvation. “*The patient at the beginning of the third stage usually spent the day supported in the arms of a close relative”*. Extraocular movements were jerky or, to the contrary, slow and rigid. Deep reflexes were exaggerated but Babinsky sign was never noticed. Generalized muscle wasting became evident and fasciculation, spontaneous or evoked by tapping, were seen. Some symptoms of dementia were also observed. Even in terminal stages, they understood the Fore language and tried to accommodate the request of the examiner. A strong grasp reflex occurred as well as fixed dystonic postures, athetosis and chorea. In one case “*almost constant small involuntary movements, involving mouth, face, neck, and hands”* were seen.

Terminally, “the patient lies moribund inside her hut surrounded by a constant group of attending relatives. […] She barely moves and is weak and wasted. Her pressure sores may have spread widely to become huge rotting ulcers which attract a swarm of flies. She is unable to speak. The jaws are clenched and have to be forced open in order to put food or fluid in. […] Despite her mute and immobile state she can make clear signs of recognition with her eyes and may even attempt to smile”. 

It is worth mentioning incredibly strong support given by Fore to dying kinsmen. “The family members live with the dying patient, siblings sleep closely huddled to their brother or sister in decubitus, parents sleep with their Kuru-incapacitated child cuddled to them and a husband will patiently lie beside her terminal, uncommunicative, incontinent, foul smelling wife” [41].

## 8. Neuropathology

**Figure 11:**
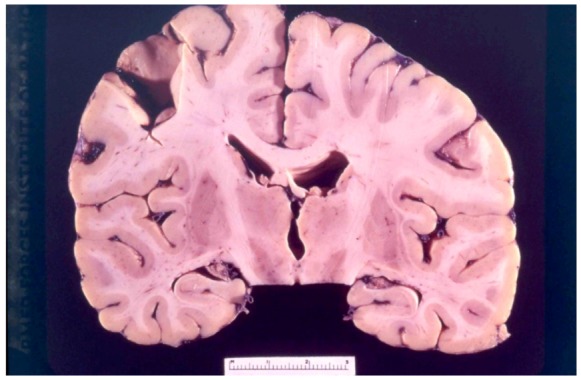
A coronal section through the kuru-affected brain. 68-3538-10. Courtesy of late D. Carleton Gajdusek.

**Figure 12:**
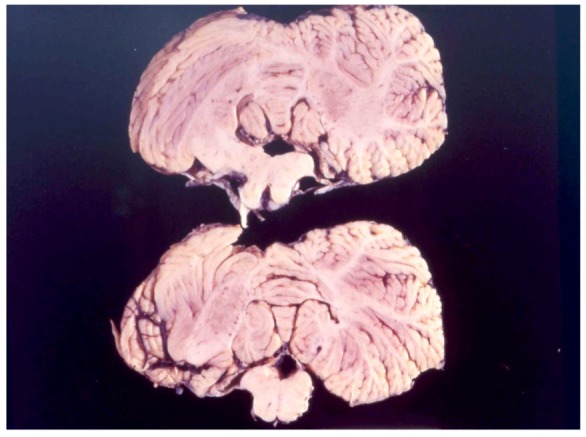
Cerebellum of the kuru-affected person. 68-3538-8. Courtesy of late D. Carleton Gajdusek.

The first systematic examination of kuru neuropathology (12 cases) was published by Klatzo *et al*. in 1959 [125,126]. Macroscopically, the brain is normal (Figure 11, Figure 12). Neuronal alterations he described were totally non-specific in nature but nonetheless sufficient to draw a parallel between kuru and Creutzfeldt-Jakob disease. 

**Figure 13:**
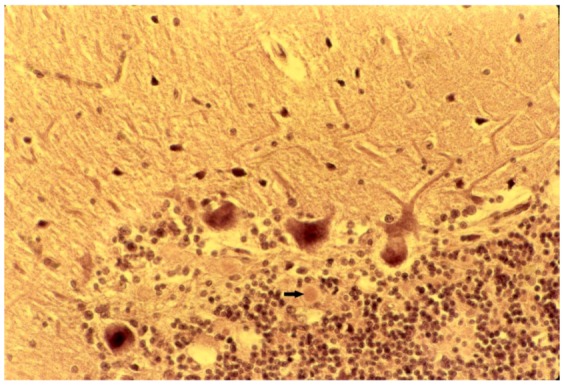
Depopulated Purkinje cell layer with a small amyloid plaque (arrow). Haematoxylin and eosin.

**Figure 14:**
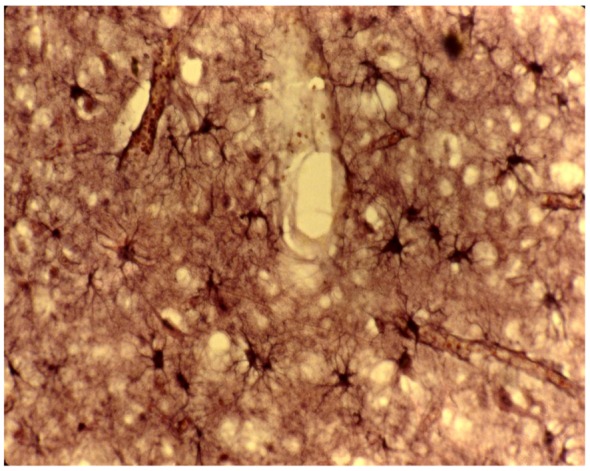
Proliferation of reactive astrocytes. Cajal Gold sublimate.

Neurons were shrunken and hyperchromatic or, pale with dispersion of Nissl substance or contained intracytoplasmic vacuoles (Figure 5) similar to those already described in scrapie. In the striatum, some neurons were vacuolated to such a degree that they looked “moth-eaten”. Neuronophagia was observed. A few binucleated neurons were visible and torpedo formation was noticed in the Purkinje cell layer, along with empty baskets that marked the presence of degenerated Purkinje cells (Figure 13). In the medulla, neurons of the vestibular nuclei and the lateral cuneatus were frequently affected; the spinal nucleus of the trigeminal nerve and nuclei of VIth, VIIth, and motor nucleus of the VIth cranial nerves were affected less frequently while nuclei of the XIIth cranial nerve, the dorsal nucleus of Xth cranial nerve and nucleus ambiguous were relatively spared. In the cerebral cortex, the deeper layers were affected more than the superficial layers, neurons in the hippocampal formation were normal. In the cerebellum, the paleocerebellar structure (vermis and flocculo-nodular lobe) was most severely affected, and spinal cord pathology was most severe in the corticospinal and spinocerebellar tracts. Astro- (Figure 14) and microglial proliferation was widespread; the latter formed rosettes and appeared as rod- or amoeboid types or as macrophages (gitter cells). Myelin degradation was observed in 10 of 12 cases. Interestingly, the significance of vacuolar changes was not appreciated by Klatzo *et al*. [125,126], but “*small spongy spaces*”, were noted in 7 of 13 cases studied by Beck and Daniel [107,108,109,110,111].

**Figure 15:**
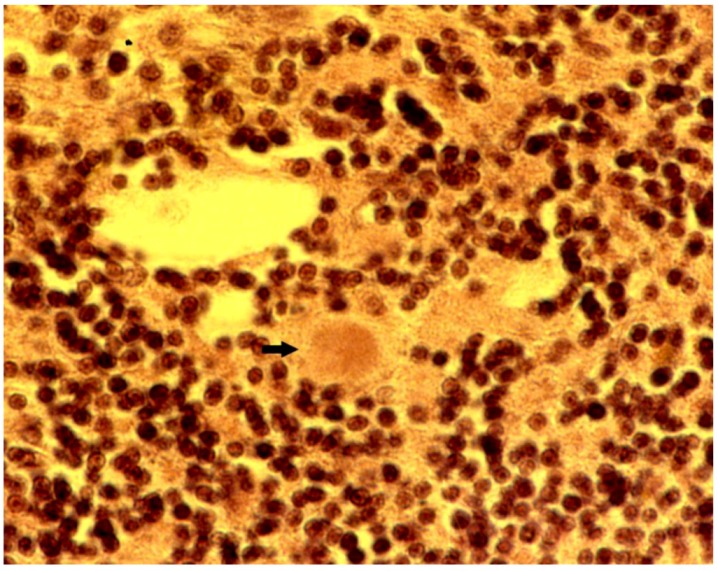
Amyloid plaque (arrow) in the granular cell layer of the cerebellum. Note small spikes at the margin of the plaques. Haematoxylin and eosin.

**Figure 16:**
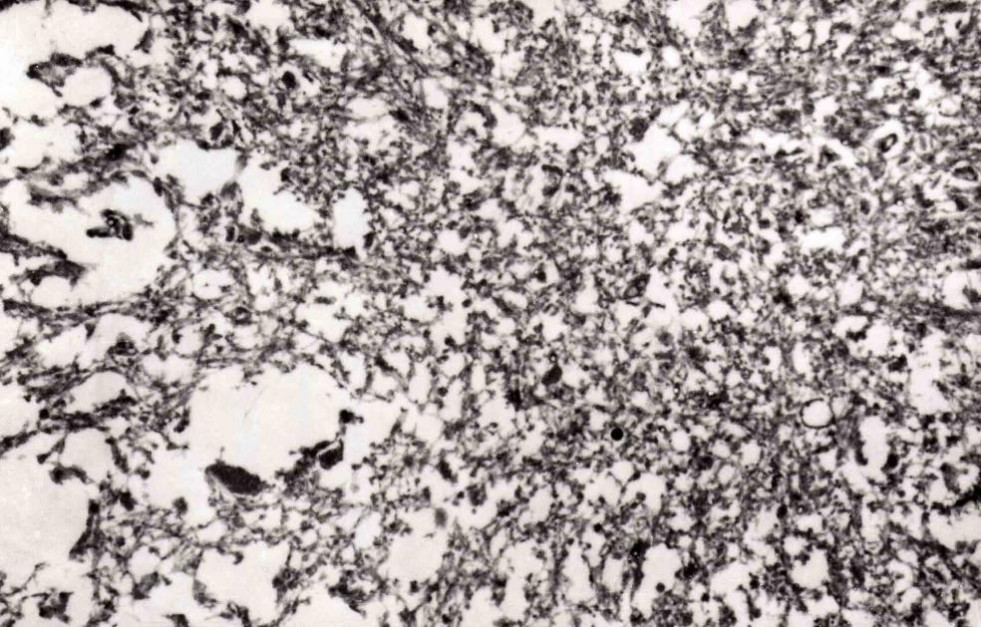
An electron micrograph of a kuru plaque retrieved from a paraffin block. Original magnification, × 10,000.

The most striking neuropathologic feature of kuru was the presence of numerous amyloid plaques found in 6 of 12 cases studied by Klatzo *et al*. [125,126], and in “about three quarters” of the 13 cases of Beck and Daniel [107,111]; they became known as “kuru plaques” [127,128,129,130,131,132,133]. These measured 20–60 μm in diameter, were round or oval and consisted of a dark-stained core with delicate radiating periphery surrounded by a pale “halo” (Figure 15, Figure 16). Kuru plaques were most numerous in the granular cell layer of the cerebellum, basal ganglia, thalamus, and cerebral cortex in that order of frequency. Kuru plaques are metachromatic and stain with PAS, Alcian blue, and Congo-red, and a proportion of them are weakly argentophilic when impregnated according to Belschowsky or von Braunmühl techniques. Of historical interest, another unique disease reported by Seitelberger [134] as *“A peculiar hereditary disease of the central nervous system in a family from lower Austria”* (in German: Eigenartige familiar-hereditare Krankenheit des Zentralnervensystems in einer niederoosterreichen Sippe) was mentioned by Neumann *et al*. [135] who was thus the first person to suggest a connection between kuru and GSS. Indeed, the latter was transmitted to non human primates in 1981 [136]. 

Renewed interest in kuru pathology has been provoked by appearance of a novel form of CJD, variant CJD, characterized by numerous amyloid plaques, including “florid” or “daisy” plaques – a kuru plaque surrounded by a rim of spongiform vacuoles [133]. To this end, a few papers re-evaluated historic material has been published [137]: We [138] studied by PrP-immunohistochemistry the case of a young male kuru victim of the name Kupenota from the South Fore region whose brain tissue had transmitted disease to chimpanzees, and McLean *et al*. [139,140] examined a series of 11 archived cases of kuru. In contrast to the classical studies described above, both papers stressed the presence of typical spongiform change present in deep layers (III–V) of the cingulate, occipital, enthorrinal and insular cortices, and in the subiculum. Spongiform change was also observed in the putamen and caudate, and some putaminal neurons contained intraneuronal vacuoles. Spongiform change was prominent in the molecular layer of the cerebellum, in peraqueductal gray matter, basal pontis, central tegmental area, and inferior olivary nucleus. The spinal cord showed only minimal spongiform change.

There are no ultrastructural observations on kuru in humans except those in a paper by Peat and Field [141] who described “intracytoplasmic dense barred structures” being an absolute normality [142] and “herring-bone” structures, again either the normal structure of the neuron or Hirano bodies [143]. In kuru in chimpanzees, Lampert *et al*. [144] and Beck *et al*. [112] found severe confluent spongiform change corresponded to typical membrane-bound vacuoles. Neurites showed dystrophic changes. Our studies on formalin-fixed paraffin-embedded Kuru specimens reversed to electron microscopy revealed typical plaques composed of amyloid fibrils (Figure 16).

**Figure 17:**
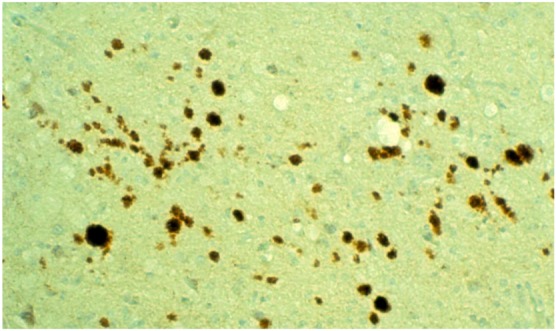
Low power micrograph showing numerous PrP^d^-immunoreactive plaques.

**Figure 18:**
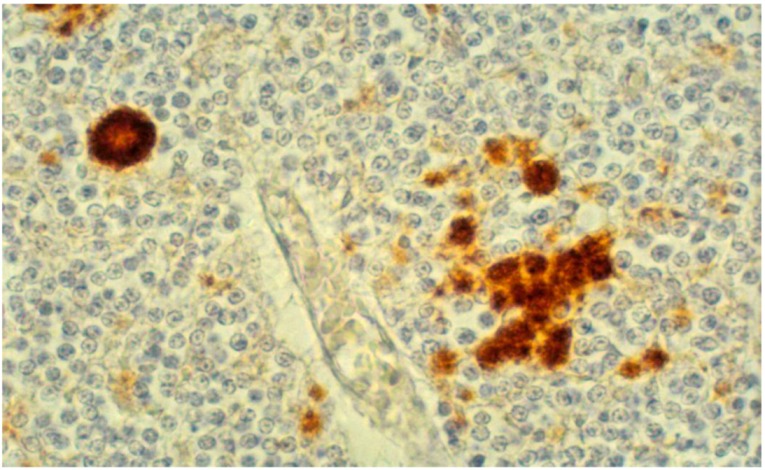
Diverse forms of PrP^d^-immunoreactive plaques.

**Figure 19:**
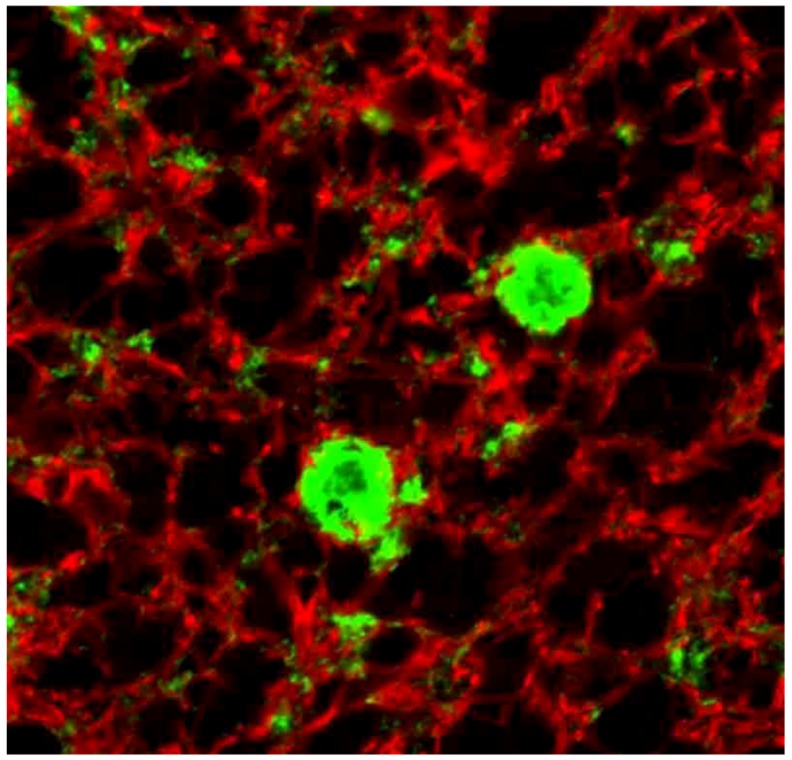
Confocal laser microscopy image of kuru plaques. Green, PrP; red, GFAP. Courtesy of Dr. Beata Sikorska, Lodz, Poland.

**Figure 20:**
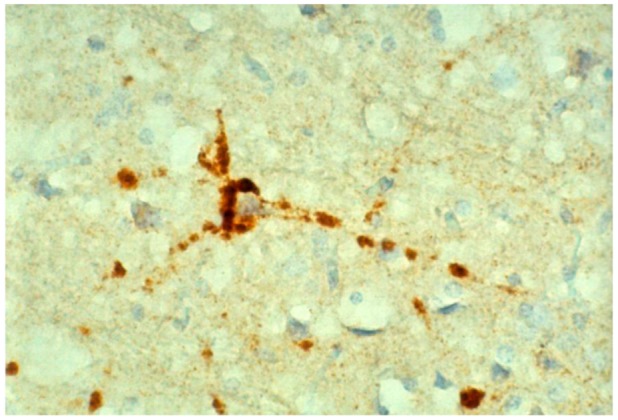
Perineuronal expression of PrP^d^.

Immunohistochemical studies revealed that misfolded PrP^d^ (d, from “disease”) was present not only as kuru plaques (Figure 17, Figure 18, Figure 19) but also in synaptic and perineuronal sites (Figure 20) [130,138], and in the spinal cord the *substantia gelatinosa* was particularly affected, as in iatrogenic CJD cases following peripheral inoculation [145]. Brandner *et al*. [146] studied one very recent case of kuru and basically confirmed the findings of Hainfellner *et al*. [138]. The latter case has been neuropathologically compared with known subtypes of CJD and it seems the most similar to type 3 129 MV type of CJD of the Collinge *et al*. [147] classification or type 2 CJD of the Parchi *et al*. [148] classification [139]. Of note, immunocytochemistry with 12F10 antibodies revealed a stronger signal than that using 3F4 anti-PrP antibodies [139].

## 9. Genetics and Molecular Biology of Kuru

Even after 40 years, the summary of the genetics of kuru written by Michael P. Alpers [85] is still valid “*it was recognized that a strong familial association of disease does not necessarily prove that the cause is genetic. Furthermore, it was hard to see how a disease so prevalent and at the same time so lethal could have become established in the population by purely genetic means, unless there was some immense associated heterozygote advantage”.* At the beginning, it was demonstrated that 2 kuru cases were 129^Met Met^ [149]. Further studies found that individuals of 129^Val Val^ and 129^Met Val^ genotype were susceptible to kuru, but those of 129^Met Met^ genotype were overrepresented in the younger age group while those of 129^Val Val^ 129^Met Val^ were overrepresented in much older age group [53,95,150,151,152,153]. In contrast, those people who survived the epidemic were characterized by almost the total absence of 129^Met Met^ homozygotes. The more recent cases studies by Lantos *et al*. [154], McLean *et al*. [140] and us [138] were all 129^Met Met^ homozygotes. Recent genome-wide studies confirmed a strong association of kuru with a SNP localized within the codon 129 but also with two other SNPs localized within genes *RARB* (the gene encoding retinoic acid receptor beta) and STMN2 (the gene encoding SCG10) [150].

The practice of endocannibalism underlying the kuru epidemic created a selective pressure on the prion protein genotype [155,156]. As in CJD, homozygosity at codon 129 (129^Met Met^ or 129^Val Val^) is overrepresented in kuru [53,95,150,151,152,153]. Furthermore, Mead *et al.* [151,152] found that among Fore women over 50 years of age, there is a remarkable overrepresentation of heterozygosity (129^Met Val^) at codon 129, which is consistent with the interpretation that 129^Met Val^ makes an individual resistant to TSE agents and that such a resistance was selected by cannibalistic rites. Another protective polymorphism G127V located in a highly conserved region of PrP was discovered by the Collinge’s group [157,158]. This 127^Val^ was not found in any of kuru patients. Because of this 129^Met Val^ heterozygote advantage, it has been suggested that the heterozygous genotype at codon 129 has been sustained by a widespread ancient practice of human cannibalism [159]. Furthermore, there is a hypothesis that extinction of Neanderthals co-existed with Homo sapiens some 45,000 to 30,000 years ago is connected to the appearance of “Kuru-like” epidemics spread by cannibalism [160,161]. Collinge *et al*. [120] suggested that the survival advantage of the *PRNP* 129^Met Met^ heterozygotes provides a basis for a selection pressure not only in Fore but also in those human populations that practiced cannibalism. Of note this was preconceived by Alpers and Gajdusek in 1965 “*In order to explain the combination of high incidence and high lethality, which at first glance might seem to entirely rule out a genetic cause unless there was an immense heterozygote advantage, we postulated that environmental change, of relatively recent origin, has given a lethal expression to a previously benign gene mutation established in the Fore population as a genetic polymorphism”* [85]*.*

**Figure 21:**
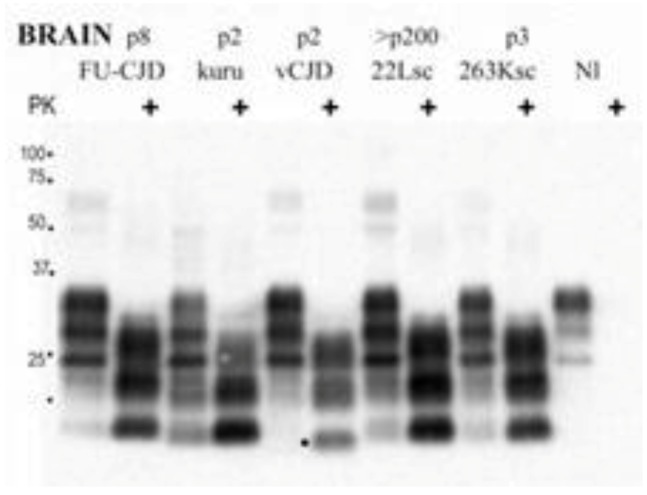
A comparison of PrP^d^ from and sCJD and vCJD. FU-CJD, Fujisaki strain of GSS; vCJD, variant CJD; 22Lsc, the 22L strain of scrapie; NI, not infected. Courtesy of Laura Manuelidis, Yale, USA.

The molecular strain typing of kuru cases was performed by the Collinge’s group [162,163]. This typing is based on the electrophoretic mobility of de-, mono- and diglycosylated bands of PrP^d^ following digestion with proteinase K [147]. The four major types of PrP^d^ were found. The human PrP^d^ type 1 and 4 occur only in individuals of the codon 129^Met Met^ of the *PRNP* gene; type 3 is seen in individuals with at least one Val at this codon and type 2 occurs in all codon 129 variants. There is another classification based on only 2 PrP^d^ types [148] and the agreement between supporters of either classification has not yet been achieved. The kuru specimens revealed type 2 (PrP^Met Met^) or 3 (129^Val Val^) PrP^d^ patterns and the glycoform ratio was similar to that of sporadic CJD but not typical for vCJD [164,165,166]. In primates inoculated with kuru and sCJD VV2 and sCJD MV 2K, the “b” pattern of pathology *i.e.*, coarser vacuoles situated in the subcortical structures and in deeper layers of the cerebral cortex was observed. PrP^d^ consisted of doublet of 20 kDa and 21 kDa. The latter notion is supported by the fact of a similar transmission rate of kuru to transgenic mice lacking mouse *PrP* gene but expressing human *PrP 129^Val Val^* gene [162,163]. In contrast, kuru was reported as not transmissible to normal wild-type mice but it was later shown that it transmits to CD-1 mice with unique clinical and neuropathological patterns in infected animals [167] (Figure 21). Of interest, the robust presence of PrP^d^ in follicular dendrititic cells in the spleen suggests a possibility of the spreading of the kuru agent via the bloodstream. Collectively, those data suggest that kuru is unique and different from either sporadic CJD or variant CJD. 

## 10. Conclusions and Speculations

Kuru, an extinct exotic disease of a cannibalistic tribe in a remote Papua New Guinea, still impacts on many aspects of neurodegeneration research. First, it showed that a human neurodegenerative disease can result from an infection with an infectious agent, and then be called a “slow virus” [100]. This discovery opened a window into the new class of human diseases including Creutzfeldt-Jakob disease, Gerstmann-Sträussler-Scheinker disease and, recently, fatal familial insomnia. Parenthetically, CJD was pointed out as a possible analogue of kuru based on non-specific neuropathological findings but Gerstmann-Sträussler-Scheinker disease was identified as linked because of the presence of numerous amyloid plaques not unlike kuru plaques. The kuru plaque became a link to Alzheimer’s disease and, as Gajdusek suggested [23], all amyloidoses share a common pathogenetic mechanism – processing of a normal protein into an amyloid deposit. This event underlies all “conformational disorders”, including pathogenetically novel classes of neurodegenerations like α-synucleinopathies, tauopathies and expanded triplet disorders. 
